# *Paeonia lactiflora* Callus-Derived Polynucleotides Enhance Collagen Accumulation in Human Dermal Fibroblasts

**DOI:** 10.3390/jfb17010056

**Published:** 2026-01-22

**Authors:** Soyoung Hwang, Seunghye Park, Jin Woo Lee, Mira Park, Le Anh Nguyet, Yongsung Hwang, Keunsun Ahn, Hyun-young Shin, Kuk Hui Son

**Affiliations:** 1 Department of Translational-Clinical Medicine, Gachon University, Incheon 21565, Republic of Korea; 2 Department of Health Sciences and Technology, GAIHST, Gachon University, 155, Gaetbeol-ro, Yeonsu-ku, Incheon 21999, Republic of Koreajwlee@gachon.ac.kr (J.W.L.); 3 Department of Molecular Medicine, College of Medicine, Gachon University, Incheon 21999, Republic of Korea; 4 Research Institute, Sphebio Co., Ltd., 501-ho, 3, Achasan-ro 11ga-gil, Seongdong-gu, Seoul 04796, Republic of Koreaceo@sphebio.com (K.A.); 5 Department of Integrated Biomedical Science, Soonchunhyang University, Asan-si 31538, Republic of Korea; 6 Soonchunhyang Institute of Medi-Bio Science (SIMS), Soonchunhyang University, Cheonan-si 31151, Republic of Korea; 7 Department of Thoracic and Cardiovascular Surgery, Gachon University Gil Medical Center, College of Medicine, Gachon University, Incheon 21565, Republic of Korea

**Keywords:** plant-derived polynucleotide, *Paeonia lactiflora*, callus culture, dermal fibroblast, collagen synthesis, ECM remodeling

## Abstract

Plant-derived polynucleotides (PNs) have emerged as promising regenerative biomolecules; however, their mechanisms remain less defined than those of salmon-derived polydeoxyribonucleotides (S-PDRNs). Here, we extracted polynucleotides from *Paeonia lactiflora* callus (PL-PN) and evaluated their biological effects on human dermal fibroblasts. PL-PN treatment increased cell viability and pro-collagen I α1 secretion. PL-PN enhanced adenosine A2A receptor expression and activated the cyclic adenosine monophosphate (cAMP)/protein kinase A (PKA)/cAMP response element-binding protein (CREB) pathway, accompanied by increased Cyclin D1 levels, retinoblastoma protein (Rb) phosphorylation, and nuclear proliferating cell nuclear antigen (PCNA) levels, indicating an accelerated G1/S transition. PL-PN also significantly reduced nuclear NF-κB localization and downregulated MMP1, MMP3, MMP9, and MMP13, suggesting attenuation of inflammatory and catabolic signaling. Furthermore, PL-PN increased TGF-β maturation, Smad2/3 phosphorylation, and the transcription of COL1A1, COL3A1, and elastin, resulting in enhanced collagen and elastin deposition. These effects are comparable to those of S-PDRN. Although the pathway specificity and in vivo relevance require further studies, our findings provide evidence that PL-PN promotes extracellular matrix regeneration via coordinated proliferative, anabolic, and anti-inflammatory actions. Thus, PL-PN represents a potential sustainable plant-based alternative to S-PDRN for dermatological regeneration.

## 1. Introduction

Skin elasticity is primarily governed by the quantity, organization, and integrity of elastin and collagen fibers in the dermal extracellular matrix (ECM). The progressive disruption of these components is recognized as a central feature of both intrinsic and extrinsic skin aging [1,2]. With advancing age, dermal fibroblasts undergo functional decline, resulting in reduced collagen biosynthesis and increased matrix degradation, which manifests clinically as reduced skin thickness, wrinkle formation, and loss of elasticity [1,2]. Consequently, strategies that sustain dermal collagen levels by enhancing fibroblast biosynthetic activity have become major targets in anti-aging research [1,2].

Polydeoxyribonucleotide (PDRN), a mixture of deoxyribonucleotide chains originally isolated from salmon DNA, has attracted considerable interest in dermatology and regenerative medicine. PDRN functions primarily by activating of the adenosine A2A receptor (A2AR) and subsequent cyclic adenosine monophosphate (cAMP)/protein kinase A (PKA)/cAMP response element-binding protein (CREB) signaling, leading to the stimulation of fibroblast proliferation, ECM production, and tissue repair [3,4,5,6]. These molecular effects are consistent with well-documented angiogenic, wound-healing, and anti-inflammatory activities in various experimental and clinical models [3,4,5,6,7,8].

Polynucleotides (PNs) are structurally related to PDRN and both consist of polymerized deoxyribonucleotide units. Earlier studies described these DNA fragments largely in terms of base-pair length, whereas more recent analyses distinguished PDRN and PN based on molecular weight and chain complexity; PDRN generally refers to short-to-medium chains, whereas PN encompasses longer or more heterogeneous nucleotide polymers [8,9,10]. Both PDRN and PN exert beneficial regenerative effects, including the promotion of wound healing, as well as antioxidant and anti-inflammatory actions, supporting their broader application in aesthetic and reparative medicine [4,6,9,10].

In addition to conventional salmon-derived preparations, alternative biological sources have been explored for producing nucleotide-based biomolecules. Marine-derived PDRN from red algae attenuates the inflammatory responses in macrophages [11], whereas PDRN extracted from sea cucumber sperm confers antioxidant and cytoprotective effects in oxidative stress-induced cell models [12]. Plant-derived PDRN is also emerging as a promising category. For example, PDRN isolated from *Panax ginseng* adventitious roots enhances skin regeneration and improves barrier function in experimental models [13]. These findings suggest that nucleic acid-based agents with PDRN- or PN-like activities can be derived from diverse nontraditional sources.

However, extracting bioactive compounds from whole plants is often limited by seasonal availability, geographical variability, and fluctuations in metabolite content, which can result in inconsistent yield and quality of the final ingredient [14,15,16]. To overcome these limitations, plant cell and tissue culture systems—such as callus and suspension cultures—have been developed as robust biotechnological platforms to produce the standardized secondary metabolites under controlled conditions [14,15,16]. In particular, callus cultures enable sterile, scalable, and continuous biomass generation that is largely independent of environmental constraints, and can provide extracts with reproducible biochemical profiles [14,15,16].

Species of the genus *Paeonia*, including *Paeonia lactiflora* and *Paeonia suffruticosa*, are medicines with a long history and are known to possess anti-inflammatory, antioxidant, and dermatoprotective properties [17,18,19,20]. Extracts from *P. lactiflora* were reported to improve a synthesis of type I collagen and downregulate matrix metalloproteinase (MMP) expression in fibroblasts, indicating their potential to support dermal ECM homeostasis [17,19]. Although the pharmacological properties of *Paeonia* species remain to be fully elucidated, existing studies suggest that they may serve as promising sources of bioactive compounds relevant to dermatologic and cosmetic applications [17,18,19,20]. Therefore, *Paeonia* callus-derived PN (PL-PN) is a promising candidate for the development of plant-origin PN with skin regenerative activity.

Building on previous reports that nucleotide-based biomolecules such as PDRN can activate A2AR-dependent cAMP–PKA–CREB signaling to drive cell-cycle progression and proliferation, while simultaneously modulating inflammatory pathways including NF-κB and matrix-remodeling cascades [3,4,5,6,10,21,22,23], we hypothesized that PL-PN would increase dermal collagen content through a similar multimodal mechanism in fibroblasts. Specifically, we postulated that PL-PN would activate the A2AR/PKA/CREB axis to promote fibroblast proliferation, attenuate NF-κB-regulated expression of collagen-degrading MMPs and favor pro-regenerative mediators such as a transforming growth factor-β (TGF-β), thereby shifting the balance toward enhanced collagen synthesis and reduced ECM degradation [9,10,21,22,23]. In this context, our study was designed to determine whether PL-PN induces collagen production in human dermal fibroblasts (HDFs) via the coordinated regulation of proliferation and matrix-remodeling pathways and to benchmark these effects against those of salmon-derived PDRN (S-PDRN), which served as a positive control.

## 2. Materials and Methods

### 2.1. Materials and Reagents

PDRN was procured from Millipore (Millipore Corp., 263023, Darmstadt, Germany), and PL-PN was obtained from Sphebio (Seoul, Republic of Korea). Dulbecco’s modified Eagle’s medium (DMEM), heat-inactivated fetal bovine serum (FBS), penicillin–streptomycin, trypsin-EDTA, and Dulbecco’s phosphate-buffered saline (PBS) were procured from Welgene (Daegu, Republic of Korea).

### 2.2. Isolation of PL-PN from P. lactiflora Callus

*Paeonia lactiflora* callus culture was performed using Murashige and Skoog (MS) basal medium. The medium was supplemented with 3% (*w*/*v*) sucrose, 0.05% (*w*/*v*) MES, 0.001% (*w*/*v*) NAA (auxin-type growth regulator), and 0.001% (*w*/*v*) kinetin (cytokinin-type growth regulator). The medium pH was adjusted to 5.8, and cultures were incubated at 25 °C in the dark for 2 weeks. All reagents were obtained from Duchefa Biochemie (Haarlem, The Netherlands). *P. lactiflora* callus cultured for three weeks was used as the source material for PN isolation. The harvested callus was frozen, ground in liquid nitrogen, and subjected to a standard plant DNA extraction procedure involving cell lysis, RNase treatment, organic solvent-based purification, alcohol precipitation, and ethanol washing. The resulting genomic DNA was dissolved in ultrapure water and used for subsequent processing.

For PN preparation, the purified DNA was fragmented using controlled ultrasonic shearing under predefined amplitude and pulse conditions optimized in-house. The molecular size distribution of the resulting PL-PN was observed by agarose gel electrophoresis using a 1.2% (*w*/*v*) agarose gel and a 100 bp DNA size marker (New England Biolabs, Ipswich, MA, USA). The purity of PL-PN was evaluated by measuring UV absorbance ratio at 260/280 nm and 260/230 nm using a spectrophotometer, microplate reader (Agilent, Santa Clara, CA, USA).

### 2.3. Cell Culture of Human Dermal Fibroblasts

Normal human adult primary dermal fibroblasts cells (HDF) were obtained from American Type Culture Collection (ATCC; PCS-201-012, Manassas, VA, USA) and grown in DMEM (Welgene, Daegu, Republic of Korea) supplemented with 10% heat-inactivated fetal bovine serum (FBS; Welgene) and 1% penicillin–streptomycin (Welgene). For treatment, 6 × 10^4^ cells were plated in 35 mm cell culture dishes and 1 μg/mL PL-PN or 10 μg/mL S-PDRN were treated for 72 h at 37 °C in a 5% CO_2_ incubator. All experiments were performed using cells between passages range 4 to 8, and no pretreatment was applied to the experimental procedures. Donor-specific information was provided by the manufacturer. PL-PN was dissolved in ultrapure water and further diluted in DPBS to prepare a stock solution. S-PDRN was dissolved in ultrapure water to prepare a stock solution. Unless otherwise specified, all concentrations reported in this study indicate the final concentrations in culture medium, and the final vehicle volume (ultrapure water and/or DPBS) was kept <1% (*v*/*v*).

### 2.4. Cell Viability Assay and ELISA of Pro-Collagen I Alpha 1 and cAMP

Cell viability was assessed using the Cell Counting Kit-8 (CCK-8; Dojindo, Kumamoto, Japan). Briefly, 1 × 10^3^ cells were seeded in 96-well culture plates, treated with PL-PN or S-PDRN at concentrations ranging from 0 to 100 μg/mL, and incubated for 24, 48, or 72 h at 37 °C in a 5% CO_2_ incubator. Then, 100 μL of medium and 10 μL of CCK-8 reagent were added to each well, and the plates were incubated for an additional 1 h. The optical density was measured at 450 nm using a microplate reader (Emax, Molecular Devices, San Jose, CA, USA).

For enzyme-linked immunosorbent assay (ELISA), 6 × 10^4^ HDF cells were seeded in 35 mm dishes and treated with 1 μg/mL PL-PN or 10 μg/mL S-PDRN for 72 h. After treatment, culture supernatants and cell lysates were collected to quantify pro-collagen I α1 and intracellular cAMP levels using a Human pro-collagen I α1 ELISA kit (ab210966, Abcam, Cambridge, UK) and a cAMP ELISA kit (ab323511, Abcam, Cambridge, UK), respectively, according to the manufacturers’ protocols. Absorbances were measured at 450 nm with a microplate reader (Emax, Molecular Devices).

### 2.5. Collagen, Elastin, and Immunofluorescence Imaging

6 × 10^4^ HDF cells were seeded in confocal dishes (100350, SPL Life Sciences, Pocheon, Republic of Korea) and incubated in complete culture medium. Cells were treated with 1 μg/mL PL-PN or 10 μg/mL S-PDRN for 72 h at 37 °C in a humidified incubator with 5% CO_2_. Following treatment, cells were fixed with 4% paraformaldehyde (PFA). For collagen and elastin staining, fixed cells were processed using a Picro-Sirius Red staining kit (ab150681, Abcam, Cambridge, UK) and a Verhoeff–Van Gieson elastin staining kit (VB-3019, VitroVivo Biotech, Rockville, MD, USA), respectively. Stained samples were visualized by a microscope (CKX53, Olympus, Tokyo, Japan).

For immunofluorescence imaging, HDF cells were fixed in 4% PFA and then blocked with 2% bovine serum albumin (BSA) (Sigma-Aldrich, St. Louis, MO, USA) in PBS for 1 h at room temperature (RT) to prevent non-specific binding. Cells were incubated overnight at 4 °C with primary antibodies against proliferating cell nuclear antigen (PCNA), protein kinase A (PKA), and NF-κB. After three washes with incubation buffer (2% BSA in PBS), samples were incubated for 1 h at room temperature with goat anti-mouse Alexa Fluor 488 or 594 (#A11001 and #A11005, 1:400, Thermo Fisher Scientific, Waltham, MA, USA) or goat anti-rabbit Alexa Fluor 488 or 594 (#A11008 and #A11012, 1:400, Thermo Fisher Scientific) secondary antibodies. Nuclei were counterstained with 1 µg/mL 4′,6-diamidino-2-phenylindole (DAPI; #D9542, Sigma-Aldrich). Samples were mounted on glass slides and examined using a confocal laser scanning microscope (LSM 900, Carl Zeiss, Oberkochen, Germany) equipped with a 20x oil-immersion objective. Confocal images were acquired with a scan speed of 7, a line average of 2, and a pinhole size of 1 Airy unit (AU). For quantitative analysis, fluorescence intensity was measured on a per-cell basis using ImageJ V.1.54k (National Institutes of Health, Bethesda, MD, USA). Individual cells were identified by DAPI-stained nuclei to define regions of interest (ROIs). Mean fluorescence intensity values were obtained from single optical sections to ensure consistency across samples and expressed as relative values normalized to the untreated control (Ctrl) group. Quantification was performed using single optical sections (Z-stack images were not used for intensity quantification to avoid projection bias) to ensure consistency across samples. At least 30 cells (*n* ≥ 30) from multiple random fields were analyzed per group.

### 2.6. Cell Fractionation and Western Blot Analysis

A Cell Fractionation Kit (#9038S, Cell Signaling Technology, Danvers, MA, USA, Cat# 9038) was used to separate the nuclear and cytoplasmic fractions for the analysis of NF-κB localization. For Western blot analysis, total cell lysates were prepared. Briefly, HDF treated with PL-PN or S-PDRN were lysed in RIPA buffer supplemented with protease and phosphatase inhibitors. Lysates were centrifuged, and the supernatants were collected in new 1.5 mL microcentrifuge tubes. Protein concentrations were determined using the Bradford assay. Equal amounts of protein (20 μg) were separated by 10% SDS–polyacrylamide gel electrophoresis and transferred onto polyvinylidene difluoride (PVDF) membranes. Membranes were blocked with 5% skim milk and then incubated overnight at 4 °C with primary antibodies against adenosine A2A receptor (A2AR; ab3461, 1:1000, Abcam, Cambridge, UK), CREB (ab32096, 1:1000, Abcam), phospho-CREB (Ser133; ab32096, 1:1000, Abcam), Cyclin D1 (96G2; #2978, 1:1000, Cell Signaling Technology), RB (SC-74570, 1:500, Santa Cruz Biotechnology, Dallas, TX, USA), phospho-RB (Ser807/811; #8516S, 1:1000, Cell Signaling Technology), NF-κB (#8242S, 1:1000, Cell Signaling Technology), Histone H3 (#4499S, 1:1000, Cell Signaling Technology), TGF-β (#3711S, 1:1000, Cell Signaling Technology), phospho-SMAD2/SMAD3 (Ser465/467, Ser423/425; PA5-110155, 1:1000, Thermo Fisher Scientific, Waltham, MA, USA), and β-actin (#5125S, 1:4000, Cell Signaling Technology). Following washing, membranes were incubated with the appropriate secondary antibodies for 1 h at room temperature. Images were acquired using an Amersham Imager 600 (GE Healthcare, Chicago, IL, USA) or by manual chemiluminescent detection with developer and fixer solutions, with protein band signals visualized using an enhanced luminescent solution (w3653-020, GenDEPOT, Katy, TX, USA) and X-ray film (Kodak). Band intensities were normalized to β-actin and Histone H3 using ImageJ.

### 2.7. Quantitative Real-Time Polymerase Chain Reaction (PCR)

Total RNA was isolated from HDF cells using RNAiso plus (9108, Takara Bio, Kusatsu, Japan) and ReliaPrep™ RNA Cell Miniprep system (Promega, Madison, WI, USA). And isolated RNA was quantified with a NanoDrop-2000 (Thermo Fisher Scientific). cDNA was synthesized with the RevertAid H Minus First Strand cDNA Synthesis Kit (Thermo Fisher Scientific). Quantitative PCR was carried out on a CFX96™ real-time system (Bio-Rad, Hercules, CA, USA) using SYBR Green I Universal PCR Master Mix (Takara Bio) and gene-specific primers (Bioneer, Daejeon, Republic of Korea) under the following cycling conditions: initial denaturation at 95 °C for 10 min, followed by 40 cycles of 95 °C for 15 s, and 60 °C for 1 min. As the reference gene, GAPDH was used. A relative gene expression was calculated by the 2^−ΔΔCT^ method with GAPDH as the internal control. The primer sequences (Bioneer, Daejeon, Republic of Korea) for Human COL1A1 (GenBank accession no. NM_000088.4), COL3A1 (GeneBank Accession NM_000090.4), Elastin (GeneBank Accession NM_000501.4), MMP1 (GeneBank Accession NM_002421.4), MMP3(GeneBank Accession NM_002422.5), MMP9 (GeneBank Accession NM_004994.3), MMP13 (GeneBank Accession NM_002427.4), and GAPDH (GeneBank Accession no. NM_017008) are presented in Table 1.

### 2.8. Statistical Analysis

All experiments were performed in triplicate or more, and data are presented as mean values unless otherwise indicated. Results are expressed as mean ± standard deviation (SD). Statistical comparisons between two groups were conducted using Student’s *t*-test in Microsoft Excel (Microsoft Corp., Redmond, WA, USA). Graphical representations were generated using Prism (GraphPad, San Diego, CA, USA). Statistical significance was set at * *p* < 0.05, ** *p* < 0.01, and *** *p* < 0.001 for all experiments.

## 3. Results

### 3.1. Characterization of PL-PN

**Figure 1:**
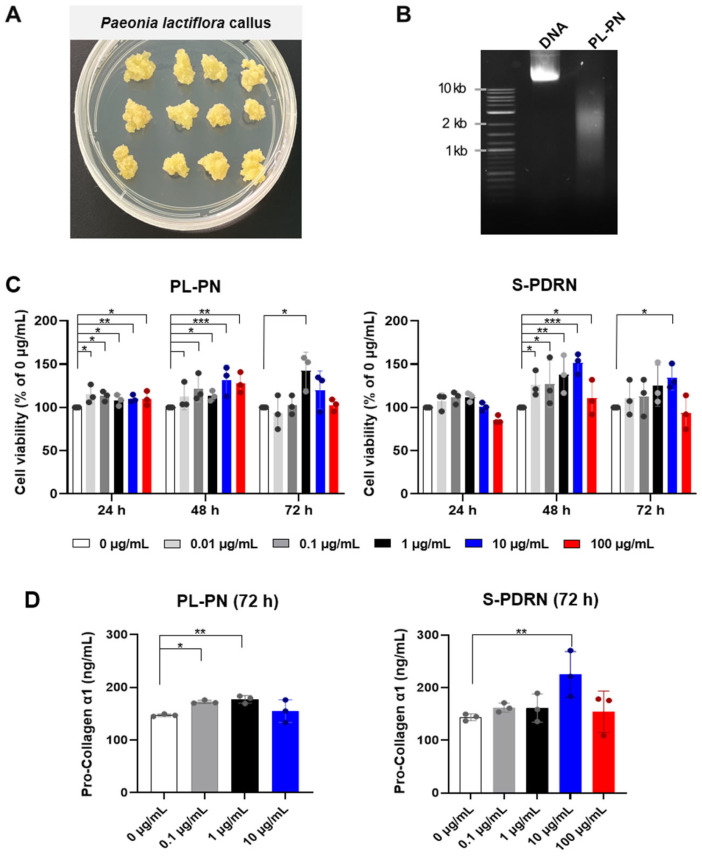
Preparation of PL-PN and its Effects on Fibroblast Viability and Pro-Collagen Production. (**A**) Three-week-old *Paeonia lactiflora* callus used for polynucleotide (PL-PN) extraction. (**B**) Agarose gel electrophoresis showing high-molecular-weight genomic DNA and fragmented PL-PN (~2 kb). (**C**) Human dermal fibroblasts (HDFs) viability treated with PL-PN or salmon-derived polydeoxyribonucleotide (S-PDRN) at 0–100 μg/mL for 24–72 h, assessed using the Cell Counting Kit-8 assay. (**D**) Secreted pro-collagen I α1 levels after 72 h of treatment with PL-PN (0–10 μg/mL) or S-PDRN (0–100 μg/mL). Data represented the mean ± SD of ≥ 3 biological replicates. * *p* < 0.05, ** *p* < 0.01, *** *p* < 0.001 vs. 0 μg/mL.

To produce PL-PN, genomic DNA was isolated from *P. lactiflora* callus cultured for three weeks (Figure 1A) and subsequently subjected to ultrasonic fragmentation. The size distribution of the resulting PN fragments was examined using agarose gel electrophoresis. As shown in Figure 1B, non-fragmented genomic DNA displayed high-molecular-weight bands exceeding 10 kb, whereas the ultrasonically treated sample exhibited a predominant band at approximately 2 kb. DNA fragmentation was consistently confined to the intended size range across multiple batches. No smearing or accumulation of low-molecular-weight fragments was observed, suggesting controlled fragmentation rather than nonspecific degradation (Supplementary Figure S1).

The purity of PL-PN was evaluated by measuring UV absorbance ratios at 260/280 nm and 260/230 nm using a spectrophotometer microplate reader (Agilent, Santa Clara, CA, USA). The PL-PN samples exhibited an A260/A280 ratio of 1.96 and an A260/A230 ratio of 1.95, indicating high nucleic acid purity with minimal protein or solvent contamination [24]. These results demonstrate that the extraction and fragmentation procedure successfully generated shorter DNA fragments suitable for PL-PN formulation.

### 3.2. PL-PN Increased Cell Viability of HDF

To evaluate the effect of PL-PN on metabolic viability and to identify a non-cytotoxic concentration range for downstream analyses, HDFs were treated with PL-PN across a wide concentration range (0–100 μg/mL) for 24, 48, and 72 h. As shown in Figure 1C, PL-PN did not induce cytotoxicity across concentrations up to 10 μg/mL, and HDFs viability was maintained at a similar level to that of the S-PDRN group. At 100 μg/mL, both PL-PN and S-PDRN showed a slight reduction in viability, suggesting that excessively high concentrations may not be optimal for subsequent experiments. Thus, the overall trend indicated that PL-PN maintained fibroblast viability within the low-to-mid dose range, supporting its suitability for further analyses.

To determine the appropriate working concentrations for downstream mechanistic and ECM-related studies, we next evaluated secreted pro-collagen I α1 levels after 72 h of treatment (Figure 1D). PL-PN increased pro-collagen I α1 production at 1 and 10 μg/mL, with the 1 μg/mL concentration producing the most consistent and reproducible elevation without signs of cellular stress. In comparison, S-PDRN exhibited its most robust pro-collagen I α1 response at 10 μg/mL.

Based on these results, 1 μg/mL PL-PN and 10 μg/mL PDRN were chosen to the working concentrations for following experiments, as they represent the lowest concentrations that reliably increased pro-collagen synthesis while maintaining cell viability.

### 3.3. PL-PN Increased A2AR/cAMP/PKA/CREB Pathway in the HDF

**Figure 2:**
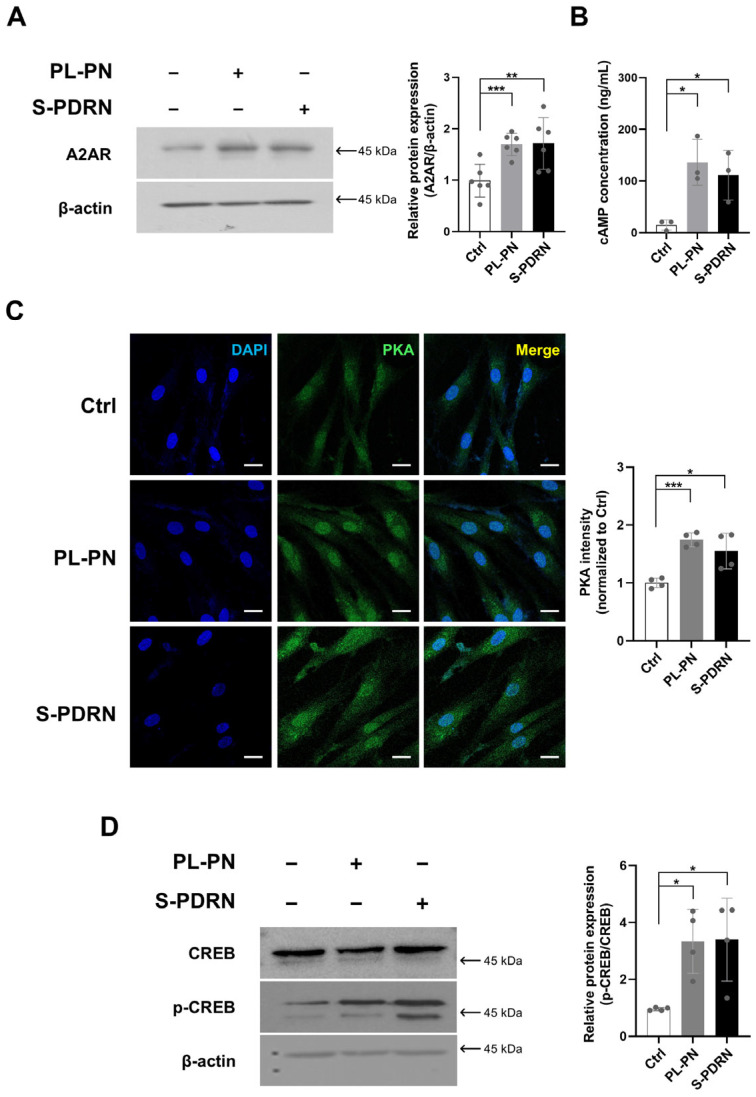
Activation of the A2A Receptor/cAMP/PKA/CREB Pathway by PL-PN. (**A**) Western blot showing adenosine A2A receptor (A2AR) expression after 72 h treatment with PL-PN (1 μg/mL) or S-PDRN (10 μg/mL). Each protein was normalized with β-actin. Data represented mean ± SD of 6 ≥ biological replicates, and the arrow indicates the expected molecular weight. (**B**) Intracellular cyclic adenosine monophosphate (cAMP) concentration measured by ELISA. Data represented three biological replicates. (**C**) Immunofluorescence staining of nuclei (DAPI; blue) and protein kinase A (PKA; green). Scale bar = 20 μm. Data represented four biological replicates. (**D**) Western blot of cAMP response element-binding protein (CREB) and phosphorylated CREB (p-CREB), normalized to β-actin. Data represented mean ± SD of ≥4 biological replicates, and the arrow indicates the expected molecular weight. * *p* < 0.05, ** *p* < 0.01, *** *p* < 0.001. vs. Ctrl.

Treatment of HDF with PL-PN (1 μg/mL) for 72 h increased A2AR protein levels compared with the untreated control. A similar increase was detected in the cells treated with S-PDRN (10 μg/mL) (Figure 2A). PL-PN treatment resulted in higher intracellular cAMP levels than those in the untreated control, and S-PDRN showed a similar pattern under the same conditions (Figure 2B). Immunostaining demonstrated increased nuclear PKA fluorescence intensity in PL-PN- and S-PDRN-treated cells compared to that in untreated controls (Figure 2C). Western blot result showed elevated levels of CREB and phosphorylated CREB (p-CREB) in cells treated with PL-PN and S-PDRN for 72 h compared to those in untreated controls (Figure 2D).

### 3.4. PL-PN Promoted G1/S Cell-Cycle Progression Markers in HDFs

**Figure 3:**
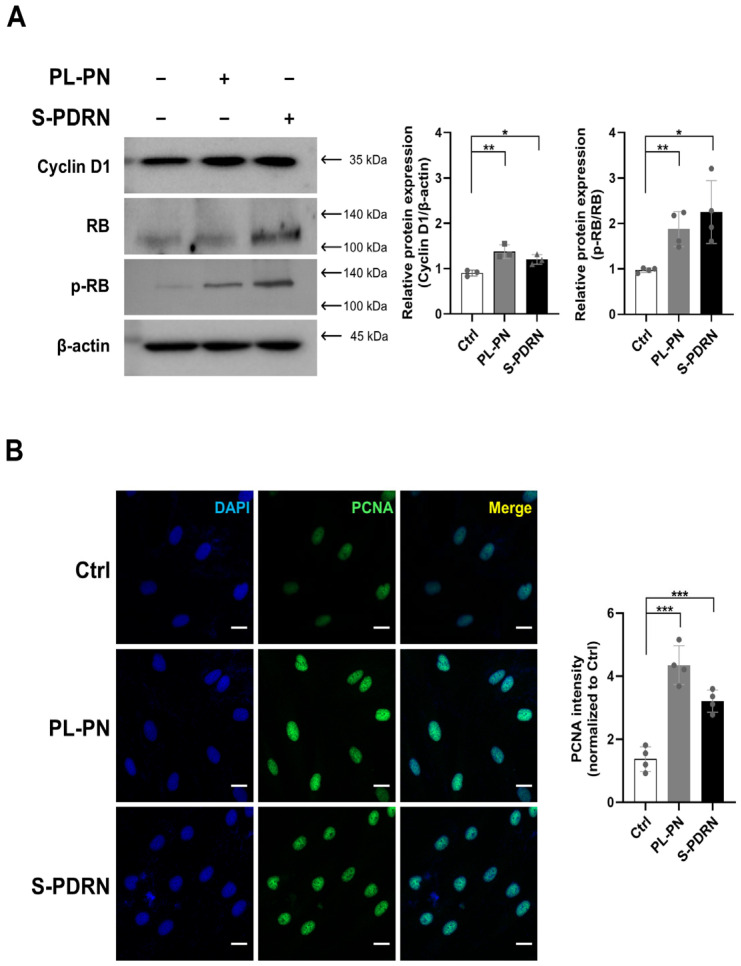
PL-PN Promoted G1/S Cell-Cycle Progression in Human Dermal Fibroblasts. (**A**) Western blot analysis of Cyclin D1, retinoblastoma protein (RB), and phosphorylated RB after 72 h treatment with PL-PN or S-PDRN. The bar graphs showed the ratio of phospho-RB to total RB protein. All Data represented mean ± SD of ≥4 biological replicates, and the arrow indicates the expected molecular weight. (**B**) Immunostaining of proliferating cell nuclear antigen (PCNA; green) with nuclear counterstain (DAPI; blue) (Scale bar = 20 μm). All Data represented mean ± SD of ≥4 biological replicates. * *p* < 0.05, ** *p* < 0.01, *** *p* < 0.001. vs. Ctrl.

To assess whether PL-PN promotes cell-cycle progression, we examined key G1/S-phase mediators [25,26]. PL-PN increased Cyclin D1 expression and enhanced the phosphorylation of retinoblastoma protein (p-Rb), indicating accelerated progression through the G1 checkpoint [25,26] (Figure 3A). Quantitative densitometry revealed that PL-PN induced Cyclin D1 and p-RB expression at levels comparable to those induced by S-PDRN.

PCNA immunostaining confirmed these findings, showing significantly increased nuclear PCNA intensity in PL-PN-treated fibroblasts (Figure 3B).

PCNA is widely used as a cell-cycle progression marker, because it is markedly upregulated at S phase to support DNA replication; thus, increased nuclear PCNA reflects active cell proliferation [27].

### 3.5. PL-PN Decreased NF-κB and MMPs in the HDF

**Figure 4:**
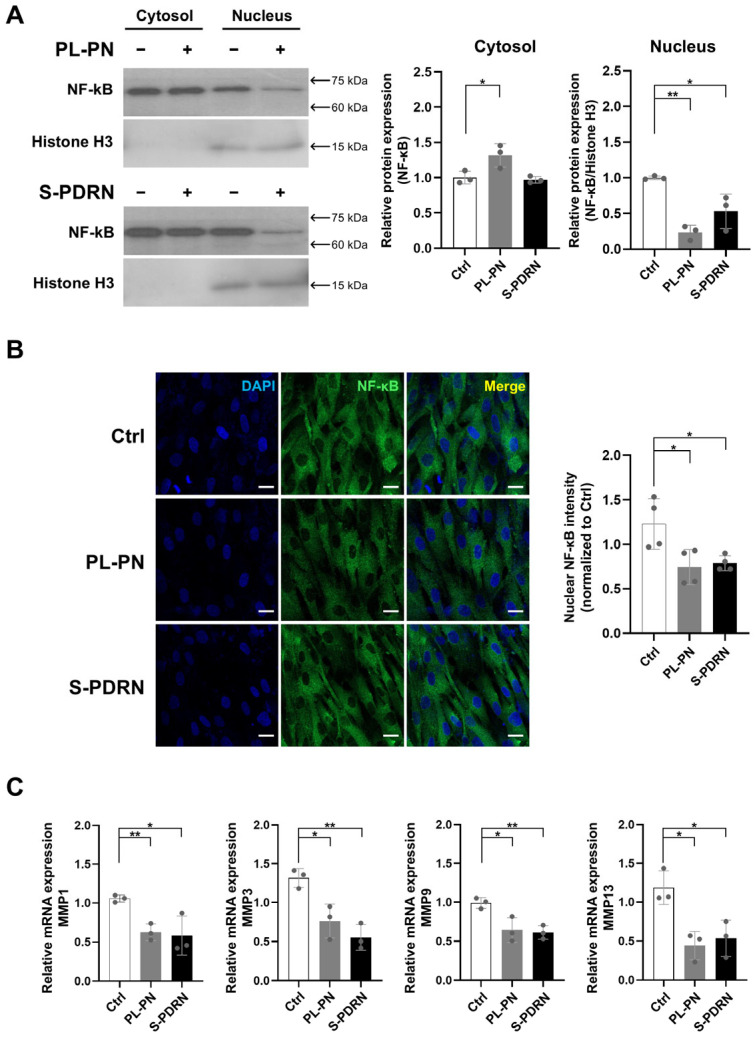
Nuclear NF-κB and MMP expression were reduced by PL-PN treatment in human dermal fibroblast cells. Data represented mean ± SD of ≥3 biological replicates, and the arrow indicates the expected molecular weight. (**A**) HDF treated with PL-PN and PDRN for 72 h, NF-κB protein expression between cytosol and nucleus was examined using a cell fractionation assay. Each protein was normalized with Histone H3. Data represented three biological replications. (**B**) Immunostaining of nucleus (DAPI, blue) and NF-κB (green) in the presence of PL-PN and PDRN at 72 h. The scale bar was 20 μm. They indicated the mean ± SD of the NF-κB nucleus intensity determined. Data represented mean ± SD of ≥3 biological replicates. (**C**) mRNA expression of MMP1, MMP3, MMP9, and MMP13 in HDF cells. Each mRNA was normalized with GAPDH as a housekeeping gene. Data represented mean ± SD of ≥3 biological replicates. * *p* < 0.05, ** *p* < 0.01 vs. Ctrl.

To investigate anti-inflammatory and anti-catabolic activity, we analyzed NF-κB localization and MMP expression. Because canonical NF-κB pathway activation requires nuclear translocation of the p65/p50 heterodimer following IκB degradation, nuclear NF-κB levels serve as a direct marker of NF-κB activation status [28]. After 72 h of treatment, PL-PN markedly reduced nuclear NF-κB levels while increasing cytosolic retention (Figure 4A), indicating the inhibition of NF-κB activation. This effect paralleled or exceeded that observed with S-PDRN. Immunostaining of NF-κB also showed that nuclear NF-κB was decreased by PL-PN (Figure 4B).

Consistent with decreased NF-κB signaling, mRNA levels of collagen-degrading enzymes MMP-1, MMP-3, MMP-9, and MMP-13 were downregulated by PL-PN (Figure 4C). Similar reductions were observed in S-PDRN-treated cells.

### 3.6. PL-PN Increased TGF-β and Smad2/3 and Increased Collagen and Elastin in the HDF

**Figure 5:**
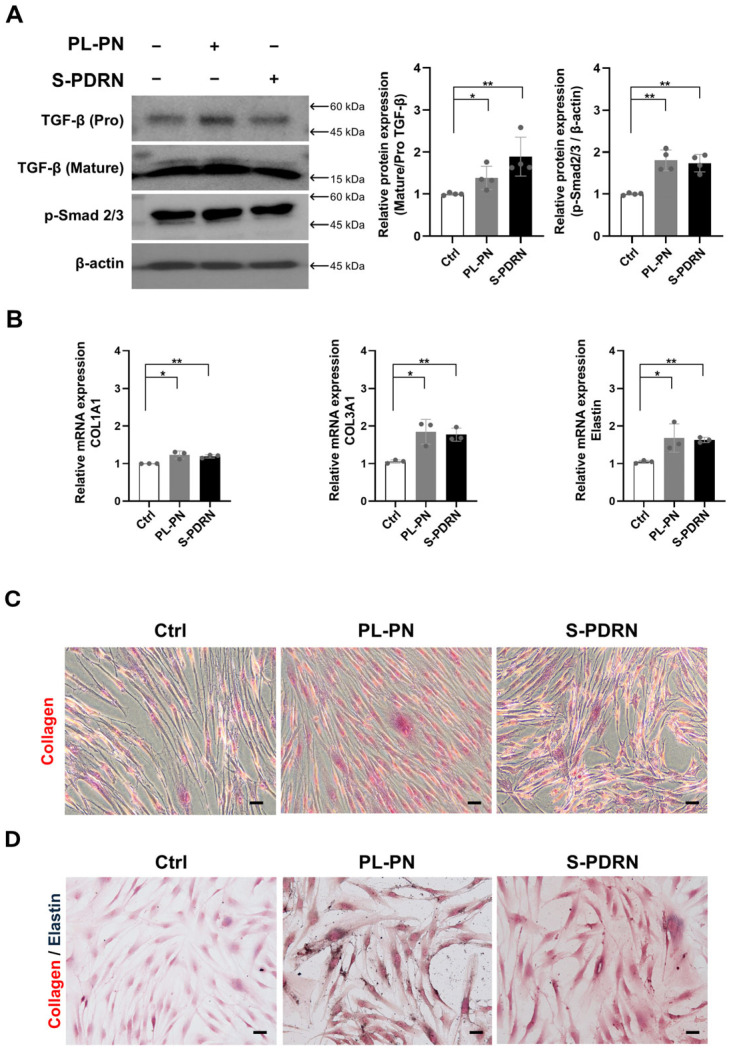
PL-PN was increased TGF-β/Smad pathway and increased collagen and elastin in human dermal fibroblast cells. (**A**) Protein expression levels of TGF-β (pro-form and mature form), p-SMAD2/3, and β-actin after 72 h of treatment. The bar graphs showed the ratio of TGF-β mature form to pro-form protein. Data represented mean ± SD of ≥4 biological replicates, and the arrow indicates the expected molecular weight. (**B**) mRNA expression of COL1A1, COL3A1, Elastin in HDF cells. Each mRNA was normalized with GAPDH as a housekeeping gene. (**C**) Sirius red stained collagen in HDF cells during 1 μg/mL PL-PN and 10 μg/mL S-PDRN treatment at 72 h. Objective: 20×; scale bar: 50 µm. (**D**) Elastin and collagen in HDF cells were stained during 1 μg/mL PL-PN and 10 μg/mL PDRN treatment at 72 h for elastin (black or blue black) and collagen (red). Objective: 20×; scale bar: 50 µm. Data represented mean ± SD of ≥ 3 biological replicates. * *p* < 0.05, ** *p* < 0.01 vs. Ctrl.

To determine whether PL-PN enhances ECM production through anabolic pathways, we assessed TGF-β and Smad activation. PL-PN increased both pro-TGF-β and mature TGF-β protein levels, along with Smad2/3 phosphorylation of after 72 h of treatment (Figure 5A). These results indicate an activation of the canonical TGF-β/Smad pathway, which is a master regulator of collagen biosynthesis [29]. In addition, PL-PN upregulated mRNA levels of COL3A1, COL1A1, and elastin (Figure 5B), confirming the transcriptional upregulation of the major ECM components. Histological assessment using Sirius Red staining revealed higher collagen deposition in PL-PN-treated fibroblasts than in the control, and levels comparable to those of S-PDRN (Figure 5C). Additionally, elastin staining showed increased elastin fiber accumulation in PL-PN-treated cells (Figure 5D). S-PDRN-treated cells displayed a similar pattern.

## 4. Discussion

Our study demonstrates that PL-PN activates multiple regenerative pathways in human dermal fibroblasts. PL-PN enhanced fibroblast proliferation, increased pro-collagen production, and stimulated A2AR–cAMP–PKA–CREB signaling, indicating that PL-PN directly modulates key mechanisms involved in ECM synthesis. These cellular responses were comparable to those observed with S-PDRN, which is known to promote fibroblast activity through similar molecular pathways [3,4,5,6,21,22,23]. However, the objective of this study was to investigate the potential of PL-PN and its regenerative mechanisms, rather than to suggest it as a direct replacement for S-PDRN.

A2AR agonism enhances collagen synthesis, angiogenesis, and tissue repair and has been translated into improved clinical outcomes in aging skin, atrophic scars, and chronic wounds [7]. CREB activation is further associated with increased transcription of genes responsible for ECM biosynthesis and cytoprotection, providing a mechanistic basis for the improved dermal density and resilience often observed following PDRN therapy [30,31,32]. The present findings suggest that, despite its plant origin, PL-PN achieves comparable phosphorylation of CREB.

Phosphorylated CREB acts as a transcriptional activator that promotes cell-cycle progression by inducing the expression of key G1/S regulatory genes. Upon phosphorylation at Ser133, CREB recruits CBP/p300 coactivators and ties up cAMP response elements (CRE) in target gene promoters [33]. This activation enhances the transcription of cyclin D1, cyclin A, and PCNA, which supports G1 progression, RB phosphorylation, and entry into the S phase [33,34]. In addition, CREB activity integrates upstream mitogenic signals from the MAPK and PI3K pathways, thereby linking extracellular stimulation to the transcriptional machinery that drives proliferation [33,34].

Consistent with this mechanism, PL-PN treatment in our study increased CREB and p-CREB levels in HDF, accompanied by cyclin D1 upregulation and enhanced Rb phosphorylation. These molecular changes indicated that PL-PN facilitates progression through the G1 checkpoint, which was further supported by increased nuclear PCNA staining. Collectively, these findings suggest that PL-PN activates CREB-dependent transcriptional programs that drive cell-cycle progression in fibroblasts.

Clinically, interventions that recover fibroblast proliferative capacity correlate with improvements in dermal thickness, elasticity, and wrinkle depth, findings repeatedly confirmed in trials of PDRN-based injectables [35].

In addition to anabolic signaling, PL-PN significantly reduced NF-κB activation and downregulated MMP expression. NF-κB is a well-characterized driver of skin aging, as its chronic activation in aged or photodamaged skin induces the expression of multiple MMPs, leading to accelerated ECM fragmentation and collagen loss [1,2,36]. Thus, the NF-κB–mediated upregulation of MMP-1, -3, and -9 is recognized as a central mechanism linking inflammation to structural dermal deterioration. Prior studies told that PDRN exhibits anti-inflammatory effects partly by suppressing NF-κB activation, primarily through A2AR-dependent signaling that increases intracellular cAMP and consequently inhibits IκB degradation, thereby preventing nuclear translocation of NF-κB [5,6]. Consistent with these findings, our study demonstrates that PL-PN reduces nuclear NF-κB levels and downregulates MMPs expression.

The cAMP–PKA–CREB axis has also been implicated in the regulation of TGF-β signaling in various mesenchymal systems. Phosphorylated CREB can directly bind to a CRE site within the TGF-β1 promoter, enhancing its transcription, thereby promoting TGF-β production and downstream Smad activation [37]. CREB has also been shown to increase TGF-β3 expression during tendon healing, further illustrating that CREB activation can drive TGF-β isoform upregulation in regenerative contexts [38].

In addition, NF-κB negatively regulates TGF-β/Smad signaling through the induction of Smad7 and the suppression of Smad2/3 phosphorylation [39]. Therefore, NF-κB inhibition can relieve this suppression, allowing more robust TGF-β/Smad activation.

Although the present study did not determine the precise upstream sequence, PL-PN treatment increased phosphorylated CREB, reduced nuclear NF-κB, and simultaneously elevated TGF-β expression and Smad2/3 phosphorylation. These findings suggest that PL-PN may enhance TGF-β/Smad signaling through a combination of CREB activation and NF-κB suppression, although additional mechanistic studies are needed to identify the dominant regulatory pathway.

Smad2/3 are the principal intracellular mediators of canonical TGF-β signaling and directly stimulate ECM formation by enhancing type I and III collagen genes transcription. Activation of Smad2/3 promotes their nuclear translocation and binding to COL1A1 and COL3A1 promoter regions, thereby driving fibrillar collagen synthesis during tissue repair and dermal remodeling [40]. Because these Smad-dependent increases in types I and III collagen represent the core structural responses of activated fibroblasts, the specific roles of these collagen subtypes are central to interpreting downstream ECM changes. Type I collagen supplies tensile strength, whereas type III collagen provides elasticity of tissue and repair ability; the coordinated upregulation of both genes reflects fibroblast activation toward matrix rebuilding rather than degradation [41]. Elastin, another essential ECM component, maintains tissue recoil properties and its restoration is considered a key determinant of functional rejuvenation in aged and photodamaged skin [42,43]. Consistent with these molecular findings, Sirius Red staining, which is specific for fibrillar collagen, reveals the degree of collagen fiber deposition and organization, whereas elastin staining indicates elastic fiber restoration; these histological readouts are standard indicators of ECM regeneration and structural recovery in fibroblast-based models [42,44].

In line with these established ECM-regenerative mechanisms, PL-PN treatment significantly increased the mRNA levels of COL1A1, COL3A1, and elastin, indicating the transcriptional activation of key matrix-forming genes. These molecular changes were accompanied by enhanced Sirius Red staining, demonstrating the increased deposition of fibrillar collagen, and more prominent elastin staining, suggesting the restoration of elastic fibers. Taken together, these findings show that PL-PN not only activates ECM-related gene expression but also drives the measurable accumulation of collagen and elastin fibers, supporting a coordinated anabolic remodeling response in HDFs.

In addition to its biological effects on fibroblasts, the plant-derived origin of PL-PN offers several practical advantages over salmon-derived PDRN. Plant cell and tissue culture platforms enable the closed, controlled production of bioactive ingredients with highly reproducible composition and without animal-derived contaminants and are increasingly used as “biofactories” for cosmetic actives [14,15,16]. Compared to animal-based biomaterials, plant-derived systems generally provide better scalability, lower risk of immune rejection, and fewer ethical or dietary concerns, which are particularly relevant for populations that avoid animal products for cultural, religious, or vegan reasons [14]. In the cosmetic and aesthetic fields, regulatory and consumer trends are shifting toward sustainable, non-animal, and environmentally friendly ingredients, and plant-based biomaterials are being actively explored as biocompatible, cost-effective, and low-impact alternatives [45]. Within this context, PL-PN represents a candidate plant-origin PN that, if future in vivo and clinical data confirm comparable efficacy and safety, could serve as a non-animal alternative to salmon-derived PDRN, while potentially improving supply stability, acceptability, and sustainability.

Although the present study did not directly compare callus culture with conventional plant cell culture systems, previous studies have described several features of callus-based production platforms that may be relevant when interpreting the origin and potential advantages of PL-PN. Callus cultures consist of actively dividing, dedifferentiated cells that often show relatively stable metabolic behavior and reduced variability compared with differentiated plant tissues [46,47]. Moreover, callus systems can be maintained under controlled environmental and hormonal conditions, which may support more consistent production of nucleic acids and other biomolecules than whole-plant extractions, which are influenced by seasonality and environmental stress [47,48]. Because callus cultures can also respond predictably to elicitation and scaled-up processes, they have been explored as reliable sources of standardized cosmetic and biomedical ingredients.

In this context, although we did not directly evaluate the differences between callus-derived PN and PN derived from other plant culture methods, the use of *P. lactiflora* calli suggests that PL-PN may benefit from some of these callus-associated characteristics, such as a potentially more uniform DNA composition, reduced contamination from phenolic compounds typically found in mature tissues, and a more stable biomass supply. However, these conclusions remain speculative and require further comparative studies. Nonetheless, the documented practical features of callus culture systems offer a conceptual rationale for exploring callus-derived PN as a plant-origin biomaterial with improved consistency and scalability.

The present study has several limitations. First, we did not perform A2AR inhibition or downstream blocking assays; therefore, although PL-PN increased CREB phosphorylation and activated the cAMP–PKA pathway, we cannot conclusively determine whether these effects are directly mediated through A2AR. Similarly, the mechanism underlying NF-κB suppression remains unclear, and further mechanistic studies, such as selective pathway inhibition, are required to clarify the precise upstream regulators. Second, our experiments were limited to in vitro fibroblast cultures, and no animal studies were conducted; thus, the in vivo relevance of PL-PN-induced ECM remodeling, anti-inflammatory activity, and potential pharmacokinetic behavior remains to be determined. Third, PL-PN exposure was assessed only for up to 72 h, and long-term or repeated-dose effects, including chronic toxicity and sustained ECM remodeling, were not evaluated. These issues warrant a systematic investigation in future studies. Fourth, although PL-PN demonstrated potent activation of proliferative signaling pathways, its long-term safety and the risk of excessive fibroblast proliferation (e.g., fibrosis) were not fully explored in an in vivo context. While the A2A receptor pathway is generally known to promote physiological homeostasis rather than uncontrolled growth, the complex interactions between PL-PN and other associated cell types in living tissue remain to be elucidated. Future studies involving long-term toxicity assessments will be essential to define the precise therapeutic window and safety profile of PL-PN. Fifth, β-actin and Histone H3 were employed as internal controls for protein normalization in our Western blot analyses. Although these proteins exhibited stable expression across all experimental groups in our study, we acknowledge that their levels can potentially vary under conditions of induced cell proliferation. While the consistent band intensities observed provided a reliable basis for our current findings, the use of total protein normalization would offer a more robust methodological approach, which we aim to incorporate in future research to further enhance quantitative stringency.

Nevertheless, the value of our work lies in demonstrating that PN can be successfully isolated from *P. lactiflora* calli and that this plant-derived PN exerts measurable proregenerative effects in HDFs, including enhanced ECM synthesis and reduced ECM degradation. If future in vivo and safety studies confirm that PL-PN exhibits biological activity comparable to that of salmon-derived PDRN without undesirable toxicity, callus-derived PN may be a cost-effective, scalable, and compositionally consistent alternative bioresource. Taken together, the present study identified PL-PN as a biologically active plant-origin PN capable of promoting ECM restoration through the coordinated modulation of fibroblast proliferation, anabolic signaling, and anti-catabolic pathways. These findings establish a foundation for further translational research exploring PL-PN as a sustainable and potentially clinically relevant candidates for dermatological regeneration.

Future studies will focus on confirming A2AR-dependent pathway specificity and directly quantifying proliferative responses using flow cytometry-based cell-cycle analysis and/or EdU incorporation. In parallel, longer-term and repeated-exposure experiments will be conducted to define a therapeutic window and evaluate safety-related endpoints, including excessive proliferation and pro-fibrotic remodeling. Finally, these findings will be validated in more physiologically relevant systems such as 3D human skin equivalents, ex vivo human skin, and in vivo models to support translational development.

## 5. Conclusions

This study demonstrates that *P. lactiflora* calli can serve as a viable source of biologically active polynucleotides capable of supporting ECM restoration in dermal fibroblasts. Although the precise upstream mechanisms and their in vivo relevance require further investigation, these findings demonstrate the regenerative potential of callus-derived PN and establish a scientific basis for the development of plant-derived PN as a sustainable alternative to conventional PDRN-based approaches.

## Figures and Tables

**Table 1:** Nucleotide sequences of qRT-PCR primer.

PCR Targets	Forward Primers (5′-3′)	Reverse Primers (5′-3′)
COL1A1	CAGGCTGGTGTGATGGGATT	CTCCATCTTTGCCAGCAGGA
COL3A1	CCACTTGGGATTGCTGGGAT	GGACCACGTTCTCCACTGAG
Elastin	TTATCCAGGGGCTGGTCTCG	GGAAAGGTAACTGCGGGGAA
MMP1	AGAGCAGATGTGGACCATGC	TTGTCCCGATGATCTCCCCT
MMP3	ACAAAGGATACAACAGGGACCAA	ACCGAGTCAGGTCTGTGAGT
MMP9	CGCGCTGGGCTTAGATCATT	TCAGGGCGAGGACCATAGAG
MMP13	CAAGATGCATCCAGGGGTCC	TCTCAGGTAGCGCTCTGCAA
GAPDH	ACCAGGTGGTCTCCTCTGAC	TGCTGTAGCCAAATTCGTTG

## Data Availability

The original contributions presented in this study are included in the article. Further inquiries can be directed to the corresponding authors.

## Supplementary Materials

The following supporting information can be downloaded at: https://www.mdpi.com/article/10.3390/jfb17010056/s1, Supplementary Figure S1. Agarose gel electrophoresis analysis of DNA fragmentation following ultrasonic treatment across independent batches. DNA samples from independently prepared batches were subjected to identical ultrasonic fragmentation conditions and analyzed by agarose gel electrophoresis. M indicates DNA size marker (100 bp ladder). PL-PN represents ultrasonically fragmented DNA from Batch 1 and Batch 2, respectively. All batches exhibited a reproducible dominant fragment size range consistent with the intended fragmentation target. Notably, no extensive smearing toward low–molecular-weight regions was observed, indicating controlled fragmentation rather than nonspecific degradation; File S1. Original Western blot images.

## Abbreviations

The following abbreviations are used in this manuscript:

A2AR	Adenosine A2A receptor
BSA	Bovine serum albumin
cAMP	cyclic adenosine monophosphate
CCK-8	Cell Counting Kit-8
CRE	cAMP response element
CREB	cAMP response element-binding protein
DAPI	4′,6-diamidino-2-phenylindole
DMEM	Dulbecco’s modified Eagle’s medium
ECM	Extracellular matrix
FBS	Fetal bovine serum
HDF	Human dermal fibroblast
MMP	Matrix Metalloproteinase
PBS	Phosphate-buffered saline
PL-PN	*Paeonia lactiflora* callus-derived polynucleotides
PCNA	Proliferating Cell Nuclear Antigen
PCR	Polymerase chain reaction
p-CREB	Phospho cAMP response element-binding protein
PFA	paraformaldehyde
PKA	protein kinase A
RT	room temperature
S-PDRN	Salmon-derived polydeoxyribonucleotide
TGF-β	Transforming growth factor-beta
