# Supplementary material for: Paeonia lactiflora Callus-Derived Polynucleotides Enhance Collagen Accumulation in Human Dermal Fibroblasts
Source: J Funct Biomater. 2026 Jan 22;17(1):56. doi: 10.3390/jfb17010056 (PMC12842499; doi:10.3390/jfb17010056)
Supplement: Supplementary file 1 [file jfb-17-00056-s001.zip › Figure S1.pdf]

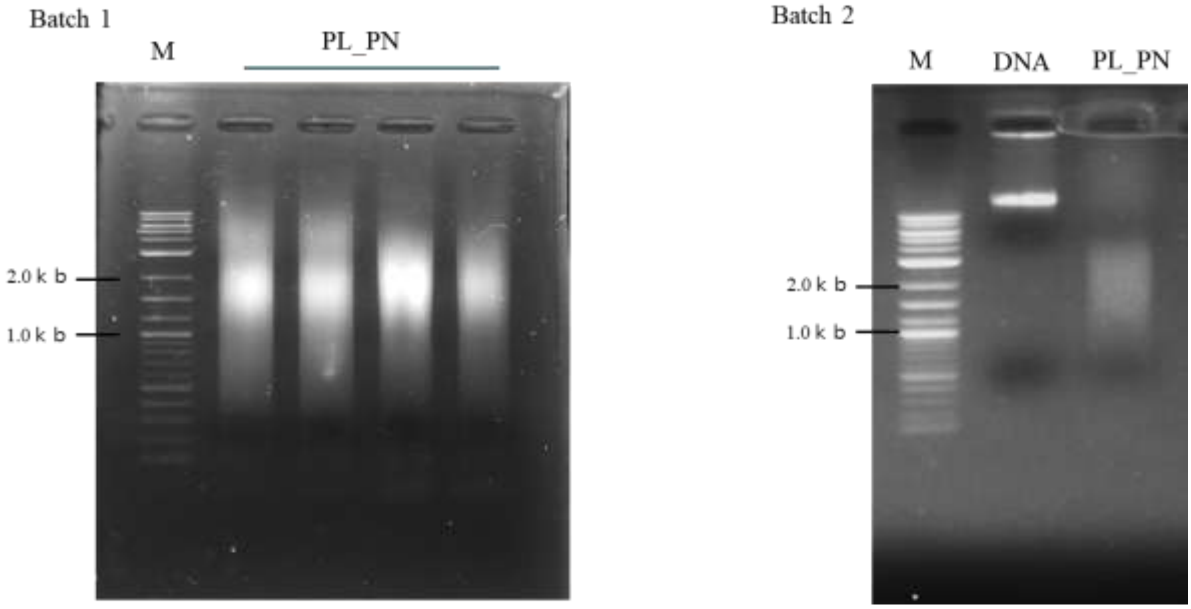

Supplementary Figure S1. Agarose gel electrophoresis analysis of DNA fragmentation following ultrasonic treatment across independent batches. DNA samples from independently prepared batches were subjected to identical ultrasonic fragmentation conditions and analyzed by agarose gel electrophoresis. **M** indicates DNA size marker (100 bp ladder). PL-PN represents ultrasonically fragmented DNA from Batch 1 and Batch 2, respectively. All batches exhibited a reproducible dominant fragment size range consistent with the intended fragmentation target. Notably, no extensive smearing toward low–molecular-weight regions was observed, indicating controlled fragmentation rather than nonspecific degradation.
