# Supplementary figures and images for: Paeonia lactiflora Callus-Derived Polynucleotides Enhance Collagen Accumulation in Human Dermal Fibroblasts
Source: J Funct Biomater. 2026 Jan 22;17(1):56. doi: 10.3390/jfb17010056 (PMC12842499; doi:10.3390/jfb17010056)

Figure 1B

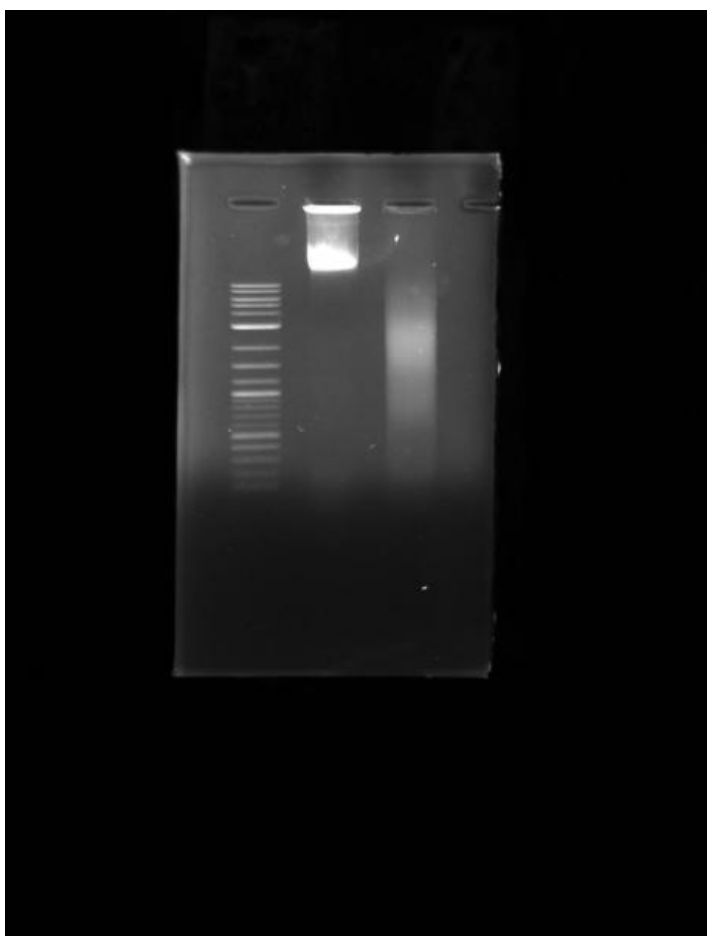

Figure 2A

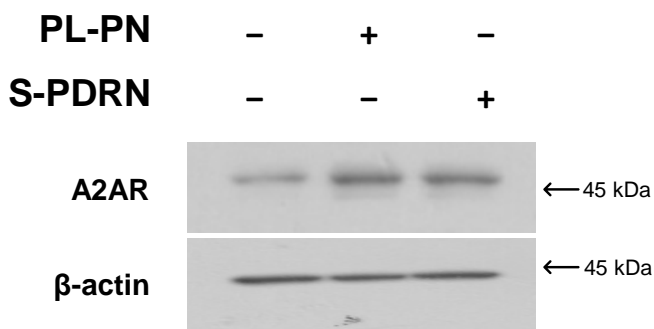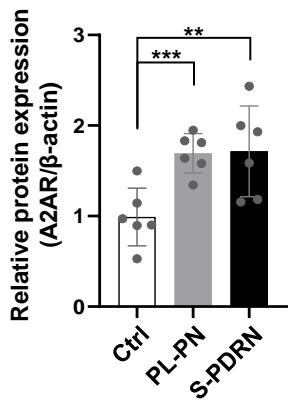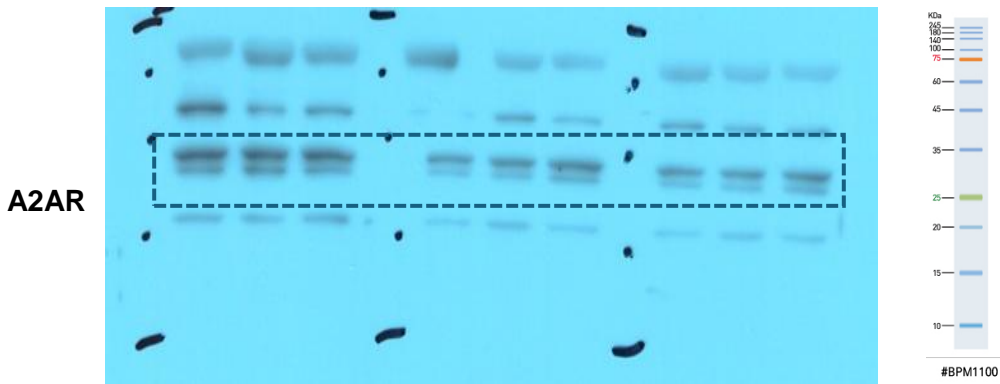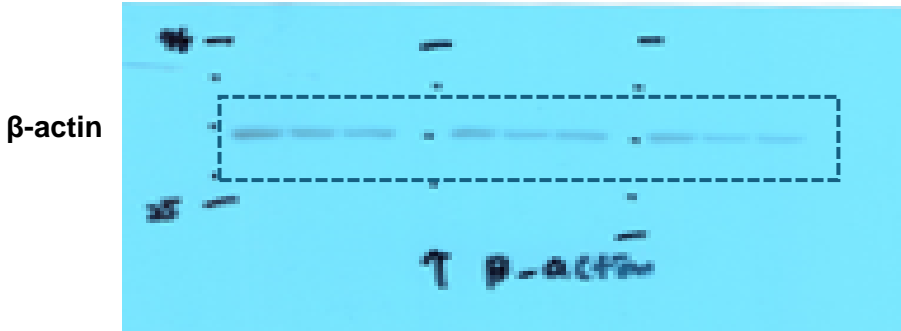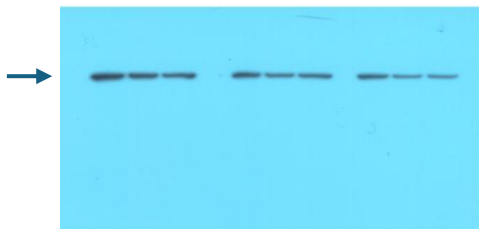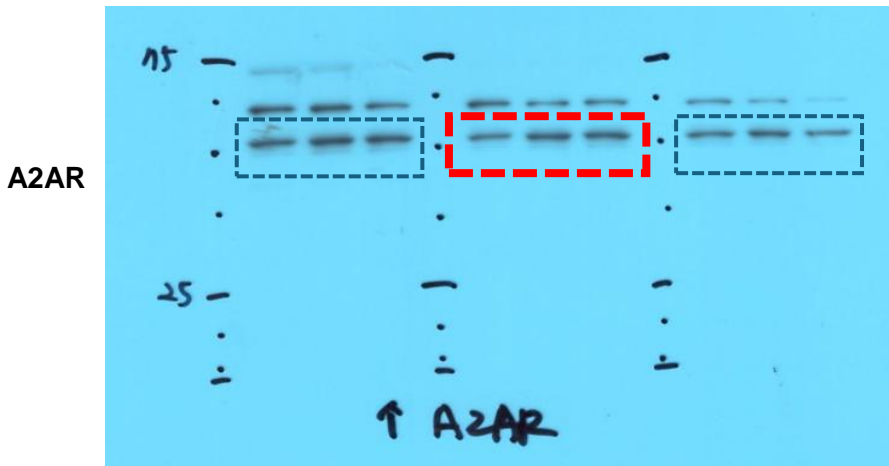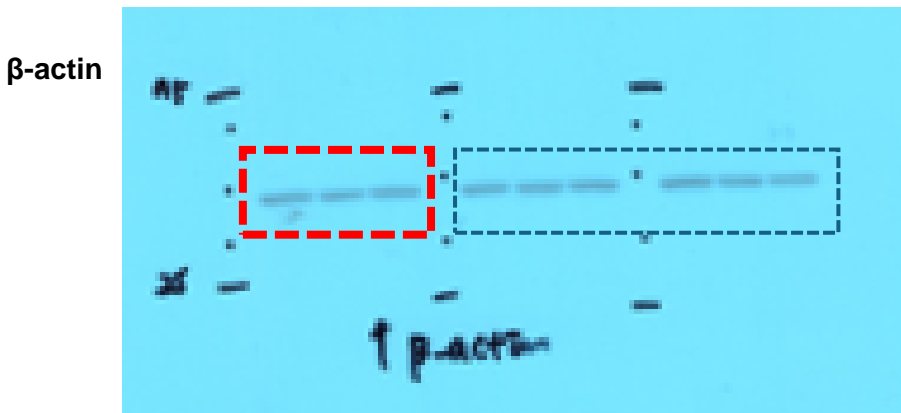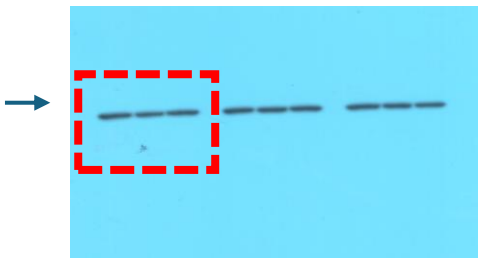

Figure 2D

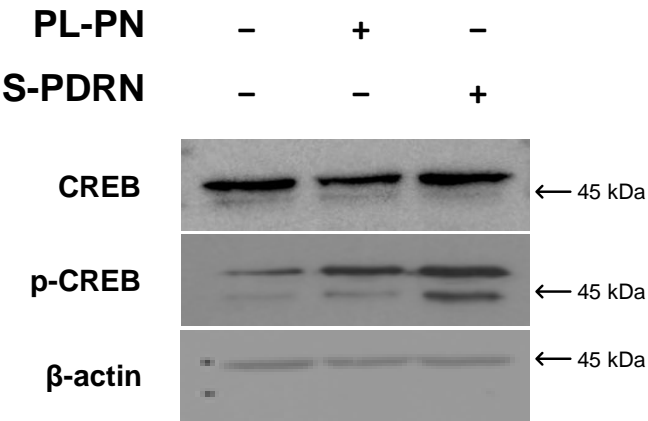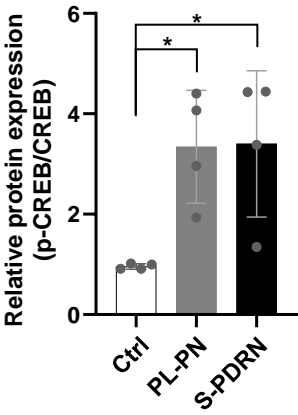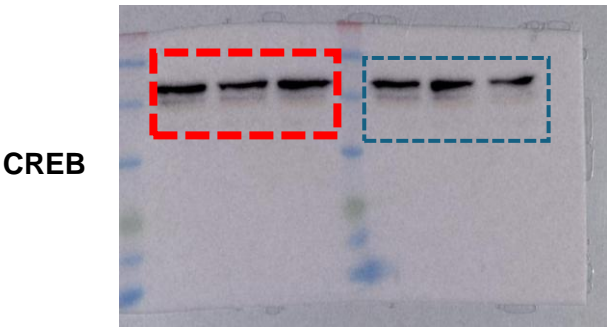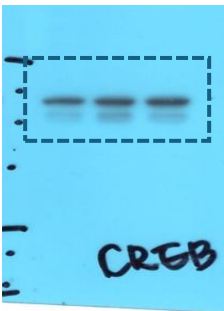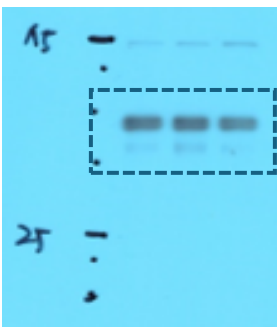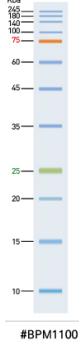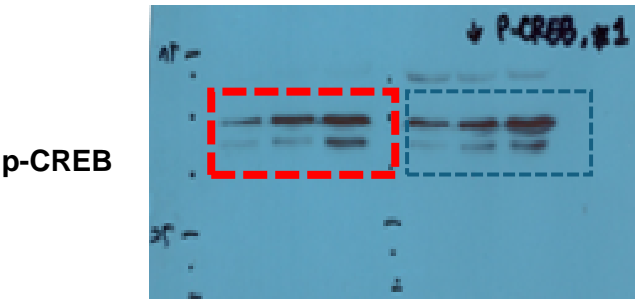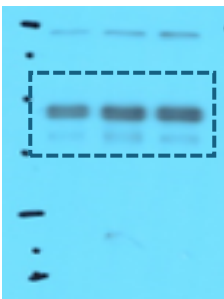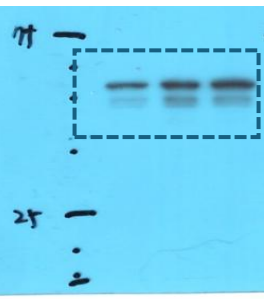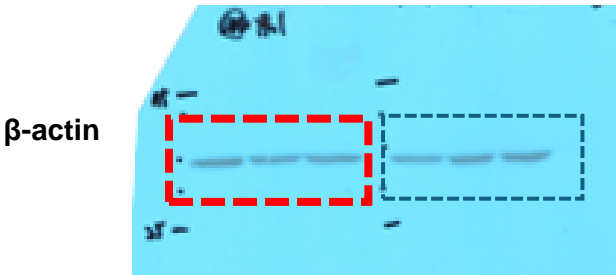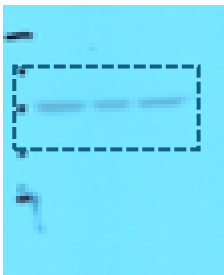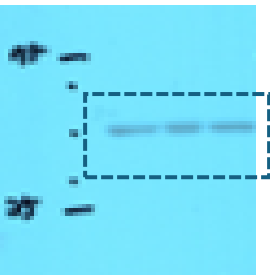

Figure 3A

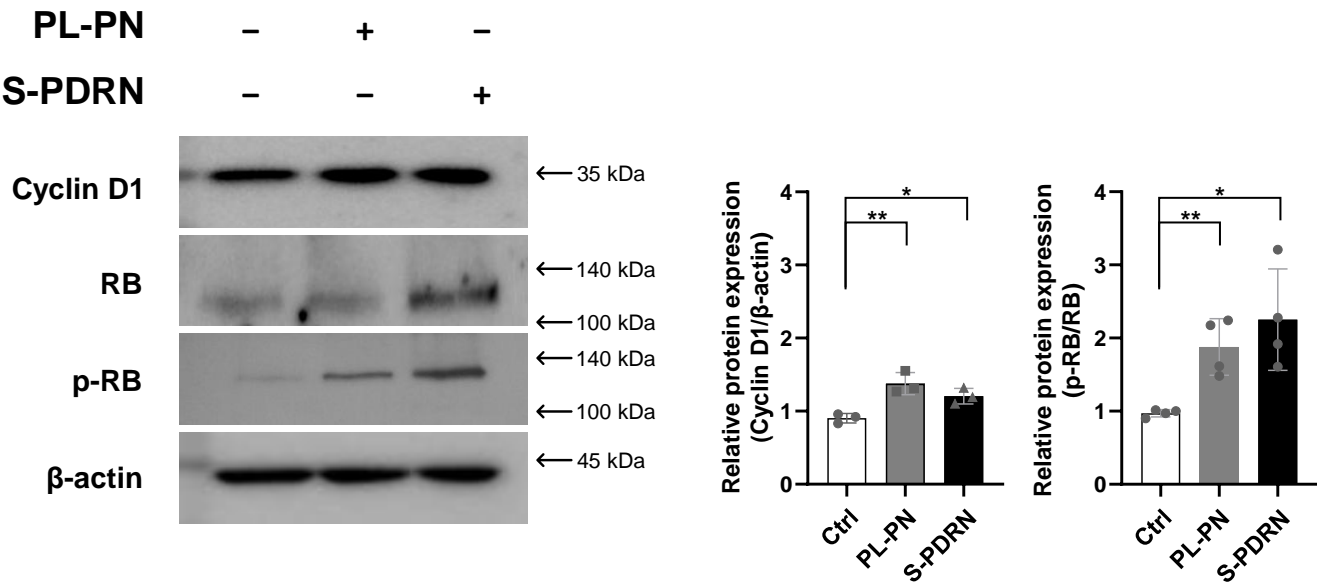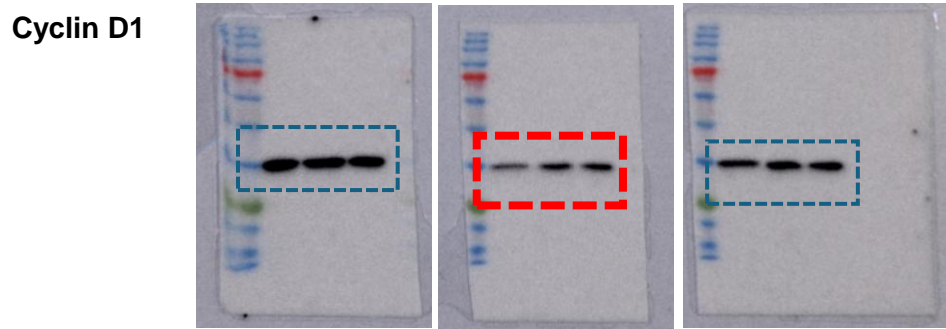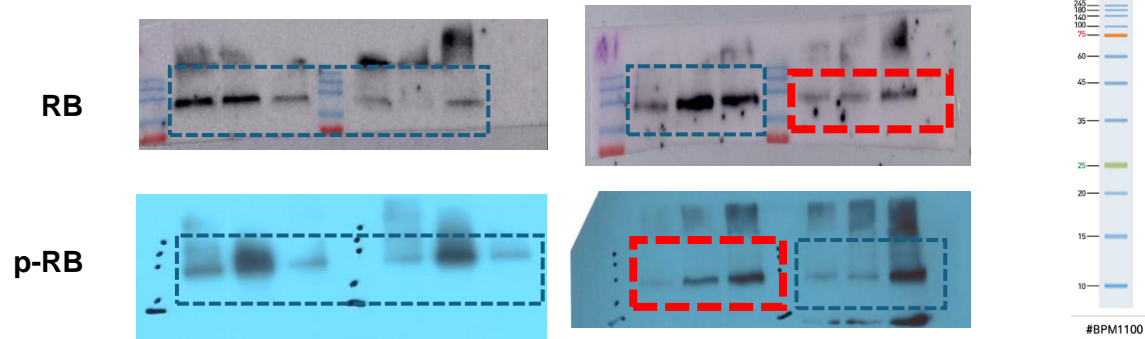

Figure 4A

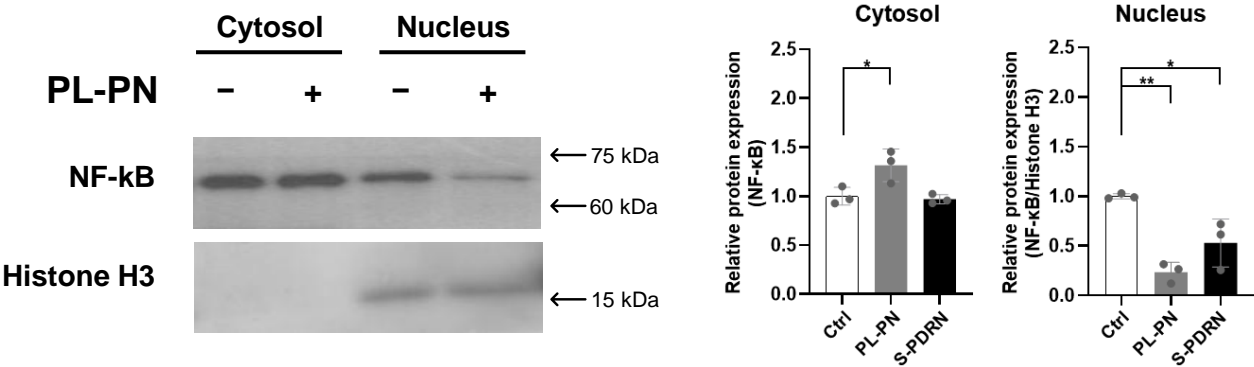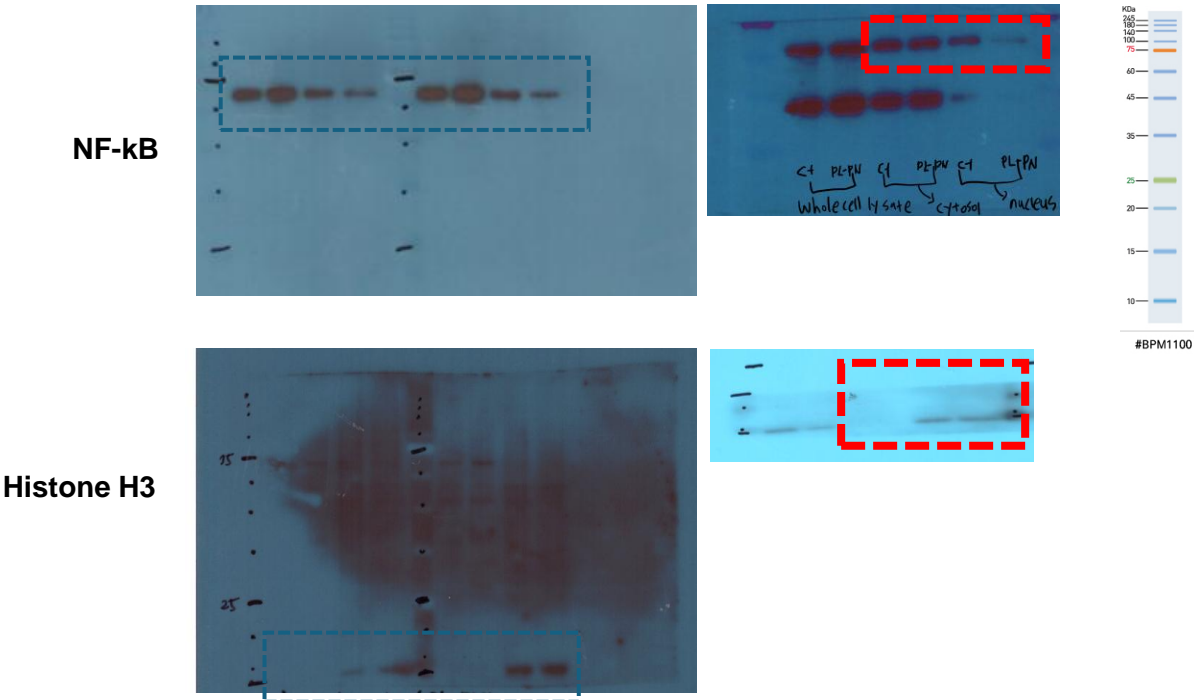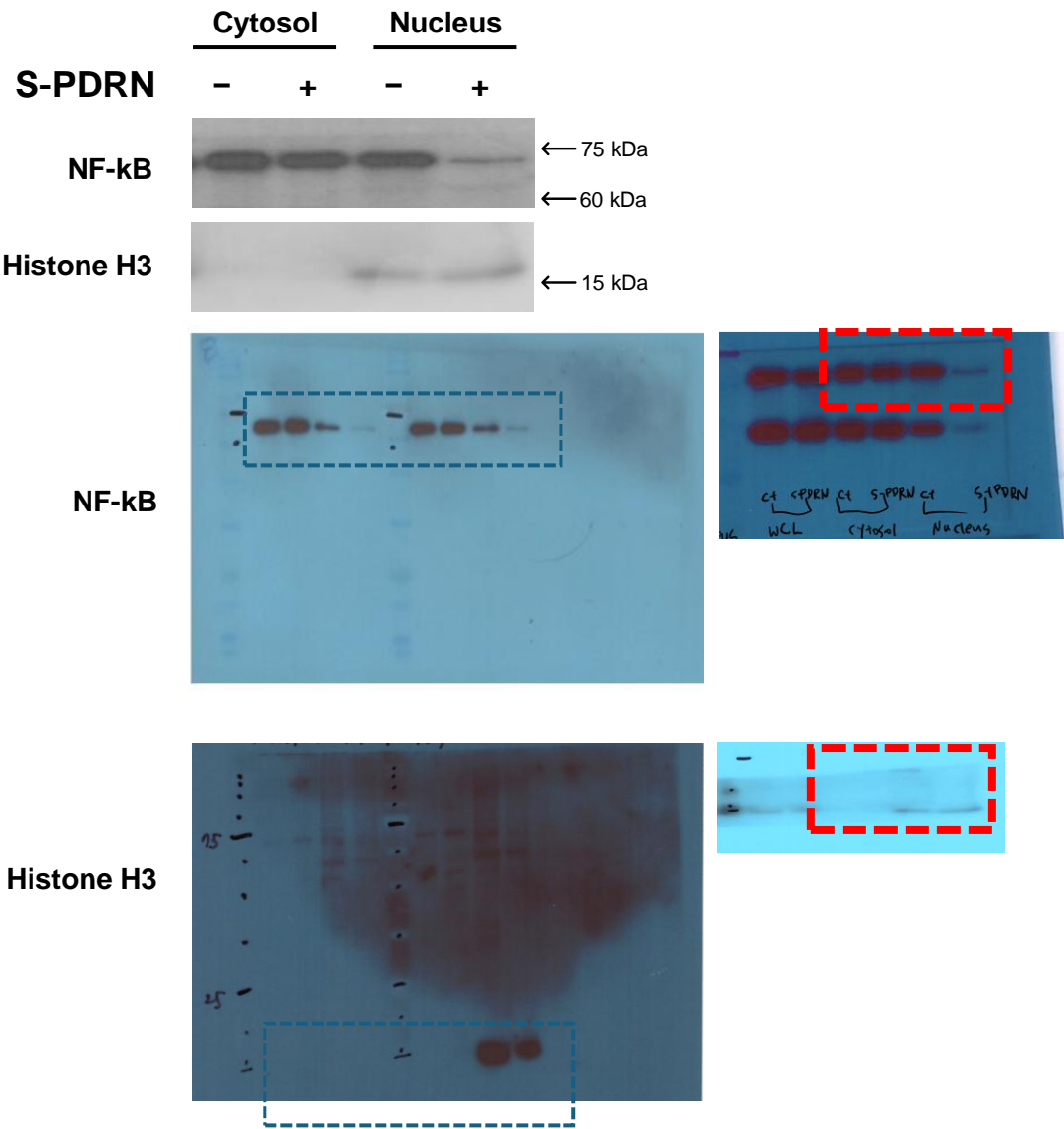

Figure 5A

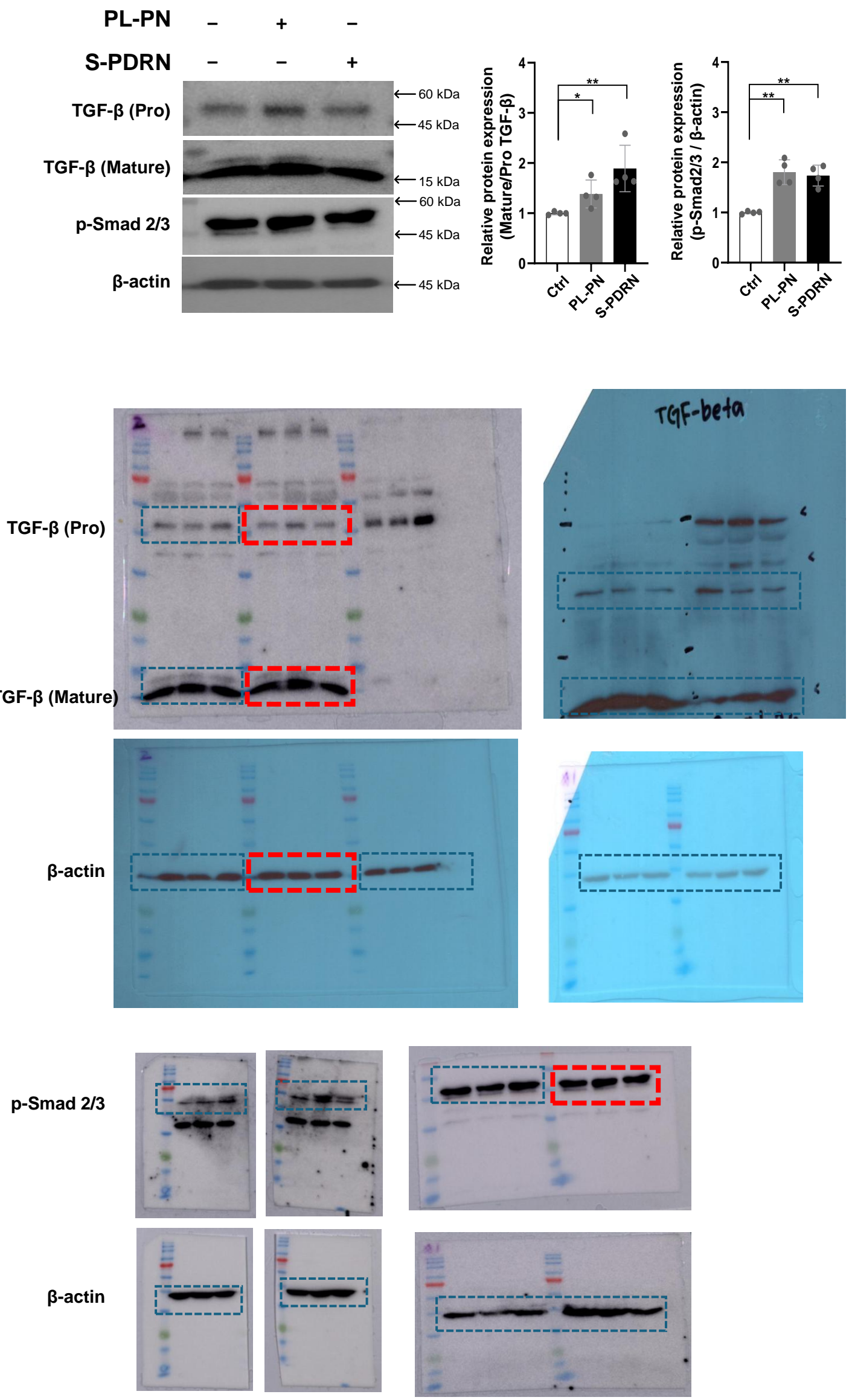

Supplement: Supplementary file 1 [file jfb-17-00056-s001.zip › File S1. Uncropped original Western blot images-revision.pdf]
